# Persistent unexplained congenital clitoromegaly in females born extremely prematurely

**DOI:** 10.1016/j.jpurol.2013.03.001

**Published:** 2013-12

**Authors:** C.E. Williams, R.S. Nakhal, J.C. Achermann, S.M. Creighton

**Affiliations:** a Institute for Women's Health, University College London Hospital, 2nd Floor North Wing, 250 Euston Road, London NW1 2PG, United Kingdom; b Developmental Endocrinology Research Group, Clinical and Molecular Genetics, UCL Institute of Child Health, 30 Guilford Street, London WC1N 1EH, United Kingdom

**Keywords:** Congenital, Clitoromegaly, Prematurity

## Abstract

**Objective:**

Unexplained clitoromegaly is a rare but well recognised feature in girls born premature. Although detected at birth, girls may re-present during childhood to paediatric urologists and gynaecologists who should be aware of this condition. The aim of the study was to describe the clinical findings and management of a series of girls presenting with persistent congenital clitoromegaly associated with prematurity.

**Materials and methods:**

This was a retrospective notes review set in a tertiary referral centre for Paediatric and Adolescent Gynaecology (PAG).

**Results:**

Eight girls with a mean age of 6 years were seen over an eight year period. In all cases a Disorder of Sex Development (DSD) had been previously excluded. The main symptoms were discomfort or concern about appearance. On examination five girls had excess skin over the clitoris and three had enlarged corporal tissue. Management included reassurance and simple measures to ease discomfort. In two cases the parents requested referral to a paediatric urologist to consider clitoral surgery.

**Conclusion:**

As survival rates for extreme prematurity improve, paediatric urologists and gynaecologists are likely to see more of these cases. Clinicians must be familiar with this condition to ensure children are managed appropriately.

## Introduction

Extreme prematurity of less than 28 weeks gestation affects approximately 1% of births. Congenital clitoromegaly in females born extremely premature is a well recognised but rare phenomenon [1,2]. Previous assumptions have been that this is a transient finding of uncertain aetiology with probable spontaneous resolution during childhood. Babies are investigated to exclude a Disorder of Sex Development (DSD) but once this has been ruled out there is little reported follow-up.

However, advances in neonatal care mean that the survival rates for very premature babies are improving and increasing numbers of girls with clitoromegaly may present during childhood to paediatric urologists and gynaecologists. Clinicians may be unaware of this condition and there is little guidance available. The aim of this study was to draw attention to this phenomenon by describing the clinical features and management of eight girls referred to our service. We also present a review of the current theories which may explain this poorly understood clinical finding.

## Materials and methods

This study took place in a tertiary referral centre for Paediatric and Adolescent Gynaecology (PAG) and DSD. The study was a retrospective review of the medical notes of all pre-pubertal girls referred with congenital clitoromegaly not due to a DSD. Notes were reviewed for details of gestation at birth, genital findings at birth, details of clinical re-presentation including genital findings, investigations and subsequent management. The study was approved as an audit by the Hospital Research and Development Committee.

## Results

Between 2003 and 2011 eight girls were referred to the paediatric gynaecology clinic with clitoromegaly not due to a DSD. The clinical features are given in Table 1. The mean age at the time of referral was 6 years (range 4–8 years). Clitoromegaly had been noted at birth in seven girls and sometime within the first two years of life in one girl. Seven girls were born at less than 27 weeks and one was born at 34 weeks. All of the girls had been previously investigated at their initial presentation and a DSD had been excluded.

The main symptoms on re-presentation to our clinic were discomfort or concern about the appearance. In two girls the mothers described intermittent enlargement of the clitoris which they interpreted as erections. There was no antenatal history to suggest in-utero androgen exposure. Although it is likely that all the mothers had antenatal steroid treatment, we could find no documentation of this. There were no other features of virilisation in any of the cases, although three of the cases also had labial adhesions, and one case had some early pubic hair (Tanner stage two). On examination five girls were found to have excess clitoral skin only whilst three girls also had palpably enlarged corporal tissue.

Pathological clitoromegaly secondary to hyperandrogenism was ruled out in all cases. Investigations included serum hormone profiles (including LH, FSH, testosterone, 17-hydroxyprogesterone, DHEAS and androstenedione), urinary steroid profiles (by GC–MS) and karyotype (Table 1). One girl had been diagnosed with central precocious puberty prior to referral to our service and was maintained on regular GnRHa (gonadotrophin releasing hormone analogue).

Six cases were managed conservatively with reassurance given in all cases. Simple advice such as the avoidance of soap in the genital area was given, and the use of emollients was recommended to relieve symptoms of discomfort such as chafing. Parents were advised that we would expect the clitoris to become less prominent as the external genitalia grow and develop as the girl enters puberty. In two cases the parents were keen to consider the option of clitoral reduction; one girl had a 2 cm clitoris and was experiencing erections and the other was troubled by the sensation from a moderately enlarged clitoral hood. They were both referred to the specialist paediatric urology service for review by the multidisciplinary team including psychology. Neither has so far proceeded to surgical reduction.

## Discussion

This is the first report describing the persistence well into childhood of clitoromegaly associated with premature birth. It is likely that the increasing identification of this finding is related to improved survival rates of extremely premature infants with the progressive improvement in neonatal care. This report is not an exhaustive clinical study of endocrinology but a retrospective notes review to bring this increasingly frequent phenomenon to the attention of relevant clinicians. It is clear that more detailed prospective research is required.

The focus of most follow-up studies in premature infants includes outcomes of growth patterns and neurodevelopment. Although transient genital ambiguity in females born prematurely has been reported in a few cases in the literature [1–4], follow-up studies on the gynaecological, pubertal and sexual development of these girls are completely lacking.

Pathological clitoromegaly, most commonly due to congenital adrenal hyperplasia (CAH) or in-utero exposure to androgens should always be excluded. CAH is an important disease to rule out as it can result in a life-threatening salt-losing crisis, and it requires life-long monitoring and treatment. If the karyotype is 46XX and CAH has been ruled out, other very rare causes of pathological clitoromegaly include aromatase deficiency or ovotesticular DSD. In all these conditions, there are normally other signs of virilisation such as rugose scrotal-like labia and posterior labial fusion. In such cases, investigations should include ultrasound imaging (to confirm the presence of female internal structures and lack of inguinal gonads), and a full hormone profile (serum and urinary). Ovotesticular DSD should be ruled out by Anti-Müllerian Hormone (AMH) (Müllerian Inhibitory Substance, MIS) measurement. (This cohort of girls were seen over several years when AMH measurement was not readily available in our hospital, and therefore was not measured as part of the standard work-up. Of note, none of the girls developed any virilisation at puberty making ovotesticular DSD extremely unlikely). If ovotesticular DSD is suspected, a histological assessment of the gonads should also be performed.

The initial diagnosis of isolated clitoromegaly in neonates is based upon a clinical impression which can be very subjective. This can lead to both over-diagnosis with unnecessary investigations and significant stress for parents. In an attempt to set objective measurements for clitoral size, Litwin et al. established normal standard graphs for clitoral length of infants born at 30–41 weeks gestation in relation to birth weight and gestational age [5]. Nevertheless, objective measurements in extreme premature female infants are still lacking.

The underlying mechanism of isolated congenital clitoromegaly is not understood although there are several hypotheses to explain it. One popular theory is that the lack of vulval fat and labial oedema in preterm girls makes the clitoris appear relatively larger [6]. Other theories suggest that the clitoromegaly is secondary to high circulating levels of androgens although the exact cause of this is not established. The foetal zone of the adrenal cortex persists until after 40 weeks gestation, irrespective of the gestation at delivery. This zone produces large amounts of circulating steroids, mainly dehydroepiandrosterone (DHEA) and its sulphate (DHEAS), which may cause partial virilisation in preterm girls [3,6,7]. Consequently the interpretation of steroid hormone profiles can be more complicated. An alternative hypothesis is that an exaggerated surge in luteinising hormone and re-programming of the ovary results in subsequently high circulating levels of androgens [2,6]. It is also postulated that higher, more prolonged synthesis or release of kisspeptin from the brain or placenta could contribute to the elevated gonadotrophin and androgen levels [2,6]. As these cases have shown persistence well into childhood, the clitoromegaly may not be explained by any of these hypotheses. However, the excess skin over the clitoris described in some of these girls, could be a sequela of earlier clitoral enlargement secondary to excess androgens around the time of birth.

Clitoromegaly in childhood causes a great deal of stress and anxiety for these young girls and their parents, both in terms of the perceived abnormal appearance and symptoms of discomfort, and the associated investigations. It is essential they are investigated in a timely manner and given appropriate reassurance and psychological support as required. Genital appearance continues to change and develop throughout childhood and adolescence, and into adulthood. It is probable that clitoromegaly will improve at puberty as the labia minora and labia majora grow and fat deposition occurs although there are as yet no long term studies to support this.

Whilst parents may request clitoral reduction surgery, there is evidence to suggest that such surgery in children with DSD leads to a loss of sensation and a potential detrimental impact on future sexual function. Clitoral reduction surgery in childhood is essentially cosmetic and ideally management should be focused on psychological support and general help with symptoms rather than irreversible genital surgery.

## Conclusion

As survival rates for extreme prematurity improve, paediatric urologists and gynaecologists are likely to see more girls presenting with unexplained clitoromegaly. Once DSD is excluded, management should be conservative. Long-term follow-up studies of girls born prematurely with idiopathic clitoromegaly are lacking.

## Conflict of interest statement

John Achermann is a Wellcome Trust Senior Fellow in Clinical Science (098513). There was no financial support for this study. Cara Williams, Rola Nakhal and Sarah Creighton have no conflicts of interest.

## Ethics

The study was approved as an audit by the Hospital Research and Development Committee.

## Figures and Tables

**Table 1:** Clinical features.

Patient	Age at referral (years)	Gestation at delivery (weeks)	Age first recognised (years)	Symptoms	Examination of clitoris	Other findings	Hormone profile (Androgens)	Urinary steroid profile	Karyotype	Management
1	6	26	Birth	↑ size, discomfort	2 cm	Precocious puberty	Normal	Androgen output ↑ consistent with adrenarche	46XX	GnRH for precocious puberty
2	4	26	2	Discomfort, erections	2 cm, Erectile tissue	Labial adhesions	Normal	Normal	46XX	Reassurance
3	7	25	Birth	Dislike of appearance	3 cm, prominent clitoral hood skin	Pubic hair Tanner 2	Normal	Normal	46XX	Reassurance, psychology (16 yrs)
4	6	34	Birth	Asymptomatic	Prominent clitoral skin	Labial adhesions	–	Normal	46XX	Reassurance
5	4	23	Birth	Asymptomatic	Excess tissue over clitoris	Labial adhesions	Normal	–	46XX	Reassurance
6	8	26	Birth	Discomfort, refusing to wear underwear	Mild clitoromegaly	None	Normal	Normal	46XX	Reassurance, psychology (9 yrs)
7	7	25	Birth	Discomfort, erections, avoiding swimming	2 cm, Excess clitoral hood skin	None	Normal	Normal	46XX	Referral to DSD team
8	7	27	Birth	Discomfort, ↑ size	Moderately enlarged clitoral hood	None	Normal	Normal	46XX	Referral to DSD team

## References

[1] Dumont T., Black A.Y., Ahmet A., Fleming N.A. (2009). Isolated transient neonatal clitoromegaly with hyperandrogenism of unknown etiology. J Pediatr Adolesc Gynecol.

[2] Greaves R., Hunt R.W., Zacharin M. (2008 Nov). Transient anomalies in genital appearance in some extremely preterm female infants may be the result of foetal programming causing a surge in LH and the over activation of the pituitary-gonadal axis. Clin Endocrinol.

[3] Greaves R., Kanumakala S., Read A., Zacharin M. (2004). Genital abnormalities mimicking congenital adrenal hyperplasia in premature infants. J Paediatr Child Health.

[4] Couch R., Girgis R. (2012 May). Postnatal virilization mimicking 21-hydroxylase deficiency in 3 very premature infants. Pediatrics.

[5] Litwin A., Aitkin I., Merlob P. (1990). Clitoral length assessment in newborn infants of 30 to 41 weeks gestational age. Eur J Obstet Gynecol Reprod Biol.

[6] Paul A., Deans R., Viner R., Creighton S.M. (2011). Pubertal development and sexuality in female adolescents born preterm: a review of the literature. Int J Adolesc Med Health.

[7] Midgley P.C., Russell K., Oates N., Shaw J.C., Honour J.W. (1996 Nov). Activity of the adrenal fetal zone in preterm infants continues to term. Endocr Res.

